# Nonsocial Functions of Hypothalamic Oxytocin

**DOI:** 10.1155/2013/179272

**Published:** 2013-07-07

**Authors:** Hai-Peng Yang, Liwei Wang, Liqun Han, Stephani C. Wang

**Affiliations:** ^1^Department of Pediatrics, The Fourth Affiliated Hospital, Harbin Medical University, Harbin, Heilongjiang 150001, China; ^2^Department of Obstetrics and Gynecology, The Fourth Affiliated Hospital, Harbin Medical University, Harbin, Heilongjiang 150001, China; ^3^Medical Imaging and Diagnostics, Mudanjiang Medical University, Mudanjiang, Heilongjiang 157011, China; ^4^Louisiana State University Medical School, 1501 Kings Highway, Shreveport, LA 71103-4228, USA

## Abstract

Oxytocin (OXT) is a hypothalamic neuropeptide composed of nine amino acids. The functions of OXT cover a variety of social and nonsocial activity/behaviors. Therapeutic effects of OXT on aberrant social behaviors are attracting more attention, such as social memory, attachment, sexual behavior, maternal behavior, aggression, pair bonding, and trust. The nonsocial behaviors/functions of brain OXT have also received renewed attention, which covers brain development, reproduction, sex, endocrine, immune regulation, learning and memory, pain perception, energy balance, and almost all the functions of peripheral organ systems. Coordinating with brain OXT, locally produced OXT also involves the central and peripheral actions of OXT. Disorders in OXT secretion and functions can cause a series of aberrant social behaviors, such as depression, autism, and schizophrenia as well as disturbance of nonsocial behaviors/functions, such as anorexia, obesity, lactation failure, osteoporosis, diabetes, and carcinogenesis. As more and more OXT functions are identified, it is essential to provide a general view of OXT functions in order to explore the therapeutic potentials of OXT. In this review, we will focus on roles of hypothalamic OXT on central and peripheral nonsocial functions.

## 1. Introduction 

Recent progress in studying therapeutic potential of hypothalamic nonaneuropeptide oxytocin has resumed our enthusiasm of its classical physiological functions. In the hypothalamus, OXT is predominantly expressed in two types of neurons, that is, magnocellular neurons in the paraventricular (PVN) and supraoptic (SON) nuclei, and parvocellular neurons in the parvocellular division of the PVN. In magnocellular OXT neurons, OXT and its carrier, neurophysin I, are packaged in membrane-bound large dense-core vesicles and transported down the long axons to the nerve endings in the posterior pituitary or neurohypophysis [1]. In response to increased activity of OXT neurons, OXT is released from the neurohypophysis into the blood [2] to act on variety of peripheral tissues. The magnocellular neurons and the neurohypophysis that contain OXT and its partner peptide, vasopressin (VP, antidiuretic hormone) together form the hypothalamoneurohypophysial system. Lately, OXT is found to be released into other regions of brain [3–5], likely from the terminals of the OXT neurons of the parvocellular division of the PVN and axon collaterals and distal dendrites of magnocellular neurons [6]. In addition to the hypothalamic origin, OXT is also produced in extrahypothalamic regions and peripheral tissues, for example, the retina, adrenal medulla, thymus, the pancreas, adipocytes, placenta, amnion, corpus luteum, interstitial cells of Leydig in the testis, and heart [7]. In mammals, OXT receptor (OXTR) has been identified in a broad spectrum of tissues, including myoepithelium of the mammary gland, myometrium of the uterus, endometrium, decidua, ovary, testis, epididymis, vas deferens, kidney, heart, thymus, pancreas, and adipocytes as well as the brain and spinal cord [7–9]. Thus, OXT can function in extensive central and peripheral sites. 

The functions of OXT in the brain and spinal cord cover a variety of social and nonsocial activities/behaviors [10, 11]. The social behaviors include social memory, attachment, sexual activity, maternal behavior, aggression, pair bonding, and trust. The nonsocial behaviors include brain development, learning and memory, feeding, respiration, cardiovascular activity, digestion, energy balance, thermoregulation, natriuresis, endocrine, immune regulation, pain perception, tolerance and dependence, autonomous outflow in addition to its classical role in the lactation, and parturition. Given the growing publications of OXT effects on these nonsocial behaviors and numerous recent reviews of social functions of OXT [12–17], this review constrains its scope to the nonsocial functions of hypothalamic OXT. 

## 2. Human Development

In the lifetime, OXT is extensively involved in the development of brain and peripheral organ systems. In the following, we will show the functions of OXT in brain and peripheral organ development, sexual dimorphism, and aging.

### 2.1. Brain Development

The influence of OXT on brain development emerges before parturition and peaks during mental development in adolescence. Around parturition, OXT has been shown as a messenger between mother and fetus. Shortly before the delivery, maternal OXT crossing the placenta reaches the fetal brain and induces a switch in the action of GABA from excitatory to inhibitory on fetal neurons [18], possibly due to reduction of intracellular chloride levels [19]. This action of maternal OXT can increase the resistance of fetal neurons to hypoxic insults during labor and in turn create an ideal condition for postpartum brain development. 

Following parturition, OXT becomes an essential tool for the development of mother-young attachment. In rodent pups, the first suckling episodes can activate OXT-secreting system through gastrointestinal signals, which facilitate the development of a preference for the mother [20, 21]. These early-life events can exert profound long-lasting effects on various behaviors such as fear/anxiety, stress responses, and reproductive functions [22]. OXT deficient mice fail to recognize familiar conspecifics after repeated social encounters, which can be restored by central OXT administration into the amygdala [23]. 

Learning and memory are important components of the endocrine. Excessive OXT attenuates memory consolidation and retrieval and processing of nonsocial stimuli [24]. The effect of systemically administered OXT upon delay memory retrieval is probably caused by an OXT-induced decrease in glucocorticoid release from the adrenal gland [25]. Paradoxically, central OXT is a critical mediator of social memory. Administration of OXTR antagonist into the lateral septum impaired social memory for both male juveniles and female adults [26]. This contradiction could be due to the difference in brain regions that carry the different memories. 

In humans, autism is an exemplary disease, largely due to the insufficiency of OXT or OXT actions [27]. This could appear as a decrease in plasma OXT and an increase in ineffective form of OXT derivatives [28], or a genomic deletion of the gene containing the OXTR gene and an aberrant methylation of OXTR [29]. Importantly, intranasal administration of OXT increases emotion recognition in children with autism spectrum disorders [30]. 

### 2.2. Peripheral Organ Development

OXTR is expressed at early developmental stages of mammals, such as in human amniotic fluid cells [31] and cultured mouse embryonic stem cells [32]. Thus, OXT could participate in the differentiation of the germ stem cell line at the very early stages of mammalian development [33]. It has been shown that OXT negatively modulates adipogenesis while promoting osteogenesis in both human multipotent adipose-derived stem cells and human bone marrow mesenchymal stromal cells. Interestingly, OXT can reverse ovariectomy-elicited bone loss in the mice and reduce marrow adiposity [34]. OXT stimulates the cardiomyogenesis of embryonic stem cells and adult cardiac stem cells and mediates differentiation of porcine bone marrow stem cells into cardiomyocytes [35]. OXT also has a promigratory effect on umbilical cord blood-derived mesenchymal stem cells [36]. Prolonged treatment of these cells with OXT can significantly increase the expression of connexin 43, cardiac troponin I, and alpha-sarcomeric actin when they are cocultured with cardiomyocytes [37]. Thus, OXT is potentially useful to advance embryonic stem cell development to reverse osteoporosis and repair infarction damaged cardiac tissue. 

### 2.3. Sexual Dimorphism

Between the SON and PVN, there is a sexually dimorphic nucleus, the intermediate nucleus. In adult men, this nucleus is twice as large as in adult women. In young women, this nucleus shows an initial period of decreased cell numbers during prepubertal development, necessary for the formation of sexual dimorphism [38]. In mouse hypothalamus, numbers of immunostained perikarya, OXT-immunostained axons, and the amount of OXT in females are much higher than those in males. In the limbic system, OXT neurons in the perifornical region, the lateral hypothalamus, and the ventral ansa lenticularis are mostly absent in males [39]. Moreover, the expression of OXT binding sites in the spinal cord dorsal horns and the ventromedial hypothalamic nucleus also shows the sexual dimorphism [40]. These dimorphic features possibly contribute to mental development, especially in the social cognitive domain [41] as well as gender specific central actions of oxytocin on reproduction-related functions and behaviors. 

### 2.4. Aging

Elderly individuals have dramatically different mental and physical features from the young and adults [42], such as eating and drinking, body-temperature regulation, sexual behaviors, and autonomic and endocrine responses. Most of these aging-related functional changes occur when hypothalamic integration, including the functions of OXT-secreting system, becomes undependable. As shown in aged male rats, the number of the OXT neurons and its processes decreased significantly in the medial and lateral parvocellular division of the PVN [43]. Consistently, erectile dysfunction in aged people with Parkinson's disease is treated by improving the function of dopamine-OXT pathway [44]. Moreover, the general increase in blood pressure in aged people possibly results from decreased OXT-associated vagal outflows [45, 46] and more frequent partner hugs and the resultant higher OXT levels are linked to lower blood pressure and heart rate in premenopausal women [47]. In addition, age-related physical alterations are associated with locally produced/acting OXT, since topical OXT application can reverse vaginal atrophy in postmenopausal women [48]. As evidence presented above, OXT is nevertheless a pivotal modifiable factor that can dramatically change the aging process.

## 3. Effects on Endocrine Systems

The hypothalamus and pituitary gland are important component of the endocrine system and they exert considerable influence over the functions of other endocrine glands. The hypothalamus regulates hormonal productions in the pituitary through releasing various tropic hormones, which act on the pituitary to secrete a variety of hormones that regulate growth and development, metabolism, reproductive, and endocrine functions. Nevertheless, OXT can modulate endocrine functions through interacting with these brain endocrine organs and their peripheral targets.

### 3.1. Pituitary Hormones

The pituitary is divided into three sections. The anterior lobe or adenohypophysis which constitutes the majority of the pituitary mass is composed primarily of five hormone-producing cell types: thyrotropes, lactotropes, corticotropes, somatotropes, and gonadotropes. These cells secret adrenocorticotropic hormone (ACTH), thyroid-stimulating hormone (TSH), prolactin, growth hormone, and gonadotropins-Luteinizing hormone (LH), and follicle-stimulating hormone (FSH). The intermediate lobe produces melanocyte-stimulating hormone (MSH) and endorphins, and the posterior lobe secretes VP and OXT [49, 50]. While magnocellular neurons innervate the neurohypophysis, they also innervate the median eminence where they can act on the adenohypophysis [51, 52]. OXT can also be transported into the intermediate lobe, which can be further increased by physiologic stimuli, such as suckling stimulus [53]. In rat adenohypophysis, there are receptors for OXT and VP [54] and removal of the neurointermediate pituitary significantly affects the secretion of anterior pituitary hormones [55]. Thus, OXT can function as a hypophysiotropic hormone.

In the endocrine actions, modulatory effects of OXT on ACTH secretion depend on observational conditions. OXT increases basal release of ACTH in both male and virgin female rats by acting on the PVN [56]. In addition, OXT can reduce cortisol response to stress in individuals with impaired emotional regulation abilities [57]. Thus, OXT can modulate ACTH and cortisol secretion to mobilize body function but curbs its potentially harmful consequence. In addition, OXT is also found to increase the release of prolactin [58, 59], *α*-MSH [60], LH [61], and FSH [62] as well as its own release [4, 63]. On the contrary, OXT inhibits TSH [64] and GH [65] release. Thus, OXT can extensively modulate body functions and behaviors through changing the secretion of these pituitary hormones.

### 3.2. Interaction with VP

VP is another major neuropeptide in the SON and PVN. Classical functions of VP include controlling the reabsorption of water in the kidneys, increasing arterial blood pressure, and regulating corticotrophin-releasing hormone (CRH) secretion [66]. In the central nervous system, functions of VP involve memory formation, circadian rhythm, aggression of females towards other males, temperature regulation, partner preference, sexual activity, and activation of the reward brain circuitry [67, 68]. In social functions, VP is mainly shown to play a role in male social behavior and newer studies show also an involvement in female social behavior [69, 70]. VP is secreted from the neurohypophysis in response to reductions in plasma volume and increases in the plasma osmolality as well as stimulations of many blood-borne factors, such as angiotensin II [71]. Thus, the functions of VP are closely related to OXT functions although they are sometimes in an antagonistic manner. 

The interactions between VP and OXT occur in many levels and forms. (1) The two neuropeptides are often up- and down-regulated by the same stimulus. In rats, hemorrhage and nonhypotensive hypovolemia are known to increase plasma levels of OXT and VP; arterial hypotension increases neurohypophysial release of OXT and VP [72]. Chronic hyponatremia reduces survival of magnocellular VP and OXT neurons after axonal injury [73]. (2) High concentration of OXT or VP can, respectively, activate both VP receptors and OXTR [7]. OXT can increase VP neuronal activity [74]. (3) OXT and VP share many functions, such as the antidiuretic effect via increasing aquaporin-2 in the kidney by OXT [75] and VP [76]. (4) The two hormones have also antagonistic interactions, such as the vasodilatation by OXT [77] and vasoconstriction by VP [71]. By coordinating the secretion and actions of these two neuropeptides in different spatiotemporal orders, the hypothalamoneurohypophysial system can highly adapt to the environmental challenges and keep the homeostasis of internal environment.

### 3.3. Peripheral Hormones

OXT can modulate peripheral functions by responsive release from the neurohypophysis and local sources; in turn, peripheral hormones can also modulate central OXT release. Examples have been shown in OXT regulation of the gastrointestinal and myocardial hormones. 

Insulin and cholecystokinin (CCK) are two representative gastrointestinal hormones. Increase in circulating OXT reduces insulin secretion [78] whereas intracerebroventricular OXT causes a rise of insulin levels by activation of vagal cholinergic neurons innervating pancreatic beta-cells [79]. By contrast, insulin can alter OXT levels in the hypothalamus by activating the insulin-regulated aminopeptidase [80, 81]. Similarly, CCK interacts with OXT at many levels. Administration of OXT increases plasma concentration of CCK [82]. Brain OXT facilitates CCK-elicited excitation of neurons within the nucleus of the solitary tract to further reduce meal size intake [83]. Conversely, peripheral administration of CCK can selectively activate the hypothalamic OXT neurons through CCK receptor in rats [84]. 

In cardiovascular regulation, OXT has close interactions with atrial natriuretic peptide (ANP) at both the heart and brain. OXT can directly stimulate ANP release from the atrium to promote Na^+^ and water excretion, thus reducing body water retention and suppressing VP secretion [85, 86]. In the hypothalamus, there is also a group of neurons containing ANP which inhibits VP secretion and promotes OXT release [87]. 

The modulation of OXT on peripheral endocrine organ is achieved through both hormonal role and neural approaches. For instance, OXT can maintain adrenaline levels directly by increasing sympathetic nervous tone; lack of OXT results in lower adrenalin levels [88]. Thus, OXT can modulate peripheral hormones more flexibly and efficiently.

## 4. Reproductive Functions

OXT is best known for its roles in parturition and lactation in females. In fact, other reproductive processes, such as sexual intercourse and menstrual cycle, are also modulated by OXT.

### 4.1. Menstrual Cycle

In female reproduction, menstrual cycle is one of the most important indexes of female reproductive ability. In animal experiments, OXT can modulate estrous cycle length by influencing follicle luteinization in the ovary and ovarian steroidogenesis. In the sheep, an increase in estradiol level causes intermittent increases in the frequency of the central OXT pulse generator. The high frequency pulses of OXT initiate subluteolytic levels of uterine prostaglandin F2*α* which triggers a supplemental release of luteal OXT. Luteal OXT amplifies the secretion of uterine prostaglandin F2*α* which initiates luteolysis and causes more luteal OXT to be secreted [89]. In ovulating women, plasma OXT is significantly low during the luteal phase in comparison with both the follicular and ovulatory phases. Thus, plasma OXT fluctuates throughout the menstrual cycle in normally cycling healthy fertile women. OXT can advance the LH surge; conversely, OXTR antagonists inhibit full production of the LH surge [61]. Thus, the high level of OXT before luteal phase has a role in the physiological processes of LH regulation; therefore OXT can modulate ovulation and the ensuing pregnancy. 

### 4.2. Sexual Activity

Sexual activity is a basic process of reproduction, in which the function of OXT has been extensively studied. Plasma OXT levels increase during sexual arousal in both women and men and are significantly higher during orgasm/ejaculation than during prior baseline levels [90]. In both men and women, there are very high positive correlations between OXT and electromyography intensity prior to and during orgasm [91], which is regulated by OXT from the PVN [92]. In the brain, OXT-dopaminergic neural pathways play a role not only in the erectile function and copulation but also in the motivational and rewarding aspects of the anticipatory phase of sexual behavior [93]. The success of sex depends on a close interaction of OXT with brain serotonin [94] and dopamine systems [95]. Deficits in OXT-secreting system or its interactions with brain amine systems can result in loss of libido, impotence, and lack of orgasm; conversely, overactivation of these systems may cause abnormal desire and multiple orgasms [96]. Postpartum women appear to experience a decrease in sexual interest possibly as a feature of a more generalized decrease in amygdala responsiveness to arousing stimuli, which also relates to the actions of OXT [97]. Thus, appropriate OXT levels and actions are essential to maintain the quality of sexual activity.

### 4.3. Parturition

In all placental mammals studied so far, including humans, OXT plays an important role in parturition. This OXT function is restrained by GABA inhibitory mechanism initially and then by a central opioid inhibitory mechanism in the hypothalamus; the increased inputs from birth cannel finally overcome the central inhibitory mechanisms during parturition, which allows increases in circulating OXT [98]. During labor, OXT can elicit uterine smooth muscle contraction to facilitate parturition and postpartum recovery of the uterus [99, 100]. Inappropriate OXT secretion can cause abnormal uterine contraction and preterm birth, which is largely attributable to early maturation of OXT-secreting system in the hypothalamus or excessive production of OXT in uterine decidua in late gestation [101, 102]. Thus, despite the relatively normal reproduction in OXT deficient mice [103] likely due to the compensatory effect of VP, OXT is nevertheless an essential hormone for normal parturition.

## 5. Lactation

As an extension of parturition, lactation is a necessary process for individual survival in mammals. Lactation is achieved through the milk-ejection reflex (MER) which depends on hypothalamic OXT [104, 105]. In the OXT-deficient/knockout mice, milk ejections are not available for the offspring [103]. Correspondingly, OXTR knockout mice exhibited normal parturition but demonstrated defects in lactation and maternal nurturing [106]; conditional OXTR knockout dams experienced high pup mortality [107]. In response to suckling stimulation, neural signals from the mammary glands and other sensory organs, such as the gastrointestinal tract and olfactory bulbs, reach the hypothalamus [108] and a synchronization center in the ventroposterior hypothalamic area [109]. The synchronization center activates OXT neurons in the SON and PVN simultaneously [110], leading to a bolus release of OXT and the ejection of milk from the mammary glands [111]. It is likely that the physiological processes that require pulsatile OXT secretion, such as, orgasm and ejaculation as well as tonic uterine contraction during parturition, are all under the control of the same synchronization center. Thus, studying the MER remains the best model for clarifying the regulation of OXT secretion.

In parallel with the function in maintaining mother-infant attachment and the development of mammary glands [112, 113], breastfeeding is associated with decreased risk for many early-life diseases and conditions, including otitis media, respiratory tract infections, atopic dermatitis, gastroenteritis, type 2 diabetes, sudden infant death syndrome, and obesity. In mothers, breastfeeding can decrease risk for type 2 diabetes, ovarian cancer, and breast cancer [114, 115]. Moreover, malfunctions of OXT-secreting system can cause maternal depression as well as lactation failure [116]. Since OXT is the key for successful lactation [117], further clarification of the regulation of OXT secretion during lactation has great therapeutic potential for lactation failure of nursing mothers and associated diseases.

## 6. Autonomic Functions

The autonomic nervous system or visceral nervous system is a part of the peripheral nervous system that controls visceral functions. This system consists of parasympathetic and sympathetic divisions [118]. The activity of this system influences heart rate, digestion, respiration rate, salivation, perspiration, pupil dilation, micturition, sexual arousal, and vascular tone [119, 120]. Sympathetic and parasympathetic divisions typically function in opposite but complementary fashion. In a specific physiological process, the two divisions are constitutively functioning, while with appropriate stimuli, each of the two activates alternatively, to achieve homeostasis. Nevertheless, OXT can modulate their functions through both central and peripheral OXTR.

OXT modulates autonomous functions partially by neuronal connections between the hypothalamus and autonomic function-regulating regions [121]. The PVN coordinates autonomic and neuroendocrine systems to maintain homeostasis and to respond to stress. The PVN projects directly to the sites that control cardiorespiratory function—the intermediolateral cell columns, phrenic motor nuclei in the spinal cord, rostral ventrolateral medulla, and the rostral nuclei in the ventral respiratory column in the brainstem. OXT fibers from the PVN also innervate the locus coeruleus and dorsal vagal complex in the brainstem [122] as well as lumbosacral spinal cord to areas involved in sensory processing and parasympathetic outflow to the uterus [123]. Through these pathways, OXT is directly involved in the integration of neuroendocrine and autonomic responses in the periphery and in the mediation of homeostasis-preserving responses within the central nervous system itself. 

Many effects of OXT-secreting system activation are associated with selective inhibition or excitation of sympathetic and parasympathetic nervous systems. For instance, intranasal OXT increases high frequency heart rate variability, a relatively pure measure of parasympathetic cardiac control, and decreased preejection period, a well-validated marker of enhanced sympathetic cardiac control [124]. This action is likely achieved via both increasing circulating OXT and activating brain OXT neurons via CSF [125], particularly those in the parvocellular division of the PVN that is located immediately lateral to the third ventricle. This possibility is high, since nasally applied VP causes fourfold increases in CSF VP levels by 10 min [126]. From the PVN, OXT could activate brainstem vagal neurons but inhibit gastric acid and insulin secretion, change gastric motility in response to stomach distention and to elevated osmolality, and block consumption of toxic foods [127]. Additionally, OXT could also modulate respiratory, sexual activity, micturition, and many other peripheral functions in association with the alteration of autonomic activity [7, 119, 120].

## 7. Nociception, Analgesia, and Addiction

Nociception or pain sensation is the neural processes of encoding and processing noxious stimuli. It is the afferent activity produced in the nervous system by stimuli that potentially damage tissues. Analgesia is the neural process of suppressing nociception and pain. By inhibiting the access of nociception to the thalamus and cerebral cortex, endogenous analgesia system and analgesic drugs can effectively reduce pain. In association with analgesia, inappropriate application of analgesic agents can cause addiction. 

The key brain structures relaying nociception are the periaqueductal gray [128], central nucleus of amygdala [129], and the nucleus raphe magnus [130]. These structures are under tonic regulation of OXT. Observations reveal that pain stimulation induces OXT release in the SON and that intraventricular injection of OXT antiserum inhibits the pain threshold increase induced by SON injection of l-glutamate sodium [131]. Nociceptive tooth pulp stimulation strongly elevates mRNA levels of OXT and opiate receptors in rat brain, which could result in more potent antinociception [132]. OXT exerts analgesia effects by increasing the release of opiate peptides including leucine-enkephalin, methionine-enkephalin, and beta-endorphin in the periaqueductal gray [131]. Thus, oxytocin might reduce (or attenuate) pain perception.

Substance abuse is also related to the alteration of interactions between OXT and endogenous opiate system. The common substance of abuse such as alcohol, opioid, cocaine, and benzodiazepine can influence brain reward, motivation, memory, and related circuitry directly. OXT is involved in drug addiction and withdrawal by regulating mesolimbic dopamine pathways [133]. Endogenous opioids likely reduce maternal behavior and increase novel exploration during lactation [134] by reducing OXT secretion [135, 136]. The inhibition of basal secretion can occur at the level of the neurosecretory terminals and at the cell bodies of magnocellular cells [137, 138], which occurs during milking but not by vaginal stimulation [139, 140]. 

Similar to the effect of opiates, acute alcohol consumption significantly decreases plasma OXT in nulliparous and lactating women [141]. This inhibition seems due to that ethanol increases the activity of large conductance, Ca^2+^-activated K^+^ channels as shown in isolated neurohypophysial terminals [142]. Ethanol also reduces the duration of single evoked spikes by a selective inhibition of voltage-gated Ca^2+^ currents in acutely dissociated supraoptic neurons of the rat [143]. 

Importantly, OXT can inhibit the action of addictive agents. Examples are acute cocaine-induced locomotor hyperactivity, exploratory activity, and stereotyped behavior in rodents [144], development of tolerance to ethanol, and opiates [145]. Thus, OXT has the potential to reverse the corrosive effects of long-term drugs abuse on social behavior and to inoculate against future vulnerability to addictive disorders. 

## 8. Special Sensory Organs

Many of OXT functions can be conditioned and this is largely based on the interactions between OXT-secreting system and specific sensory organs. In functional magnetic resonance imaging study, it is revealed that robust pup suckling activates much of the cerebrum, most notably the visual, auditory, and olfactory cortices [146]. Thus, enhanced sensitivity across the cortical layer during nursing likely helps the dam to perceive, process, and remember stimuli critical to the care and protection of her young. On the contrary OXT release into the blood can be conditioned to visual, olfactory, or auditory stimuli associated with suckling and feeding [147]. 

It is known that SON neurons innervate the olfactory bulb via axon collaterals [148] and increased olfactory output increases the activity of supraoptic neurons [149, 150] via projection from the olfactory bulb to the SON of the rat [151, 152]. Thus, OXT neurons and olfactory neurons can form a reciprocal neural circuit. In fact, olfactory OXT does play a critical role in brain function. For instance, vaginocervical stimulation can promote olfactory social recognition memory in female rats through the release of OXT [153] where OXTR is also identified [8, 9]. Moreover, nasal application of OXT has been associated to improving lactation failure, autism, schizophrenia, and other aberrant social behaviors [154–156] bypassing the blood-brain barrier via multiple approaches [125]. Thus, nasally applied OXT can alter OXT neuronal activity by activating olfactory system and in turn OXT release in multiple brain areas to exert therapeutic effects. 

Similarly, OXT effects are also seen in the visualization and auditory sensation. A single dose of intranasally administered OXT enhances detection accuracy of briefly presented emotional stimuli including facial emotion recognition [157], which is independent of modulations in overt visual attention [158]. OXT immunoreactive perikarya and/or fibers have been found within several nuclei in the auditory brain stem including the medial and ventral nuclei of the trapezoid body and in the cochlear nucleus [159]. OXTR distributions in singing mice support involvement of OXT system in vocal communication [160]. Moreover, there are strong associations between OXT and social processing to the auditory and vocal domain [161]. Thus, OXT could also modulate vocalization and hearing processes.

The effect of visual and auditory stimuli on conditioned MER during suckling has been studied in normal and pinealectomized lactating rats. The photic and auditory stimuli were applied to each mother for 10s every 20s during the 30 min suckling period. Both stimuli inhibit milk ejection without altering the nursing behavior. In mothers kept in complete darkness or in which the visual stimulus shone continuously during the suckling period, milk ejection was not affected [162]. It seems that the pineal gland, which receives projection of retina via suprachiasmatic nucleus, mediates an inhibitory visual reflex acting on OXT release and milk ejections. 

OXT can also modulate gustation. Absence of OXT in mice can increase daily intake of palatable sweet and nonsweet solutions of carbohydrate by selectively blunting or masking processes that contribute to postingestive satiety [163]. OXTR is expressed in taste buds throughout the oral cavity in mice and effects of OXT on taste tissue are delivered through the circulation [164]. OXT-responsive taste bud cells modulate taste signaling and afferent sensory output, which complements central pathways of appetite regulation that employ circulating homeostatic and satiety signals. The taste signals are likely delivered to the parabrachial nucleus through neurons in the nucleus of the solitary tract, which could mediate lithium chloride activation of OXT cells in the PVN and SON and the resultant aversive responses [165]. It is predictable that further study of the interactions between OXT-secreting system and these special sensory systems will lead to novel therapies that can dramatically improve the performance of OXT-secreting system as well as the quality of these special sensations.

## 9. Immune System

Immune system is essential for self-defense through destroying pathogens, neutralizing toxins, and cleaning dead cells as well as cytokine production and actions. OXT interacts with immune system during its development, homeostasis, and response to injuries. 

The interactions between the two systems have been seen in the following processes. (1) The OXT-secreting system and immune system cannot be separated histologically; they merge together to form a single immunoneuroendocrine system to carry out both the endocrine and immune functions. For instance, hypothalamic and/or pituitary cells produce many cytokines, such as interleukin (IL)-1, IL-2, IL-6, interferon-*γ*, and transforming growth factor-*β* [166]. By contrast, OXT gene and OXTR are expressed in the thymus [167] and monocytes and macrophages [168]. (2) OXT is the target of immunological cytokines and OXT can also modulate the activity of immune organs. On the one hand, prostaglandin E_2_ [169], IL-2 [170], and IL-6 [171] can increase the activity of OXT neurons in the SON and PVN or promote OXT secretion into the blood. On the other hand, OXT significantly increases peripheral blood mononuclear cell blastic response to phytohemagglutinin [172] and decreases both superoxide production and release of proinflammatory cytokines from OXTR-bearing monocytes and macrophages [168]. (3) OXT is involved in many immune diseases and has great therapeutic potentials in relieving immune injury. For example, neuronal IL-1*β* colocalizing with OXT reduces significantly in multiple sclerosis [173]. Peripheral OXT administration can inhibit atherosclerotic lesion development and adipose tissue inflammation by significantly reducing IL-6 production [174]. Continuous OXT delivery reduces inflammation and apoptosis in infarcted and remote myocardium [175], thereby improving functions of injured heart. Thus, OXT can provide great therapeutic benefit in diminishing inflammation while increasing immune responses through interacting with the immune system in this emerging immunoneuroendocrine system.

## 10. Metabolism and Energy Balance

Metabolism is chemical reactions that occur in the cells of living organisms to sustain life, including anabolism and catabolism. OXT modulates lipid, protein, and sugar metabolism by modulation of appetite and satiety, enzyme activity, cellular signals, secretion of metabolic hormones, and energy consumption [176].

OXT acts as a mediator of general and carbohydrate-specific satiety and regulator of body weight. OXTR knockdown mice show obesity and dysfunction in body temperature control when exposed to cold [177]. OXT also acts as a homeostatic inhibitor of consumption, capable of mitigating multiple aspects of ingestive behavior and energy metabolism [127]. The metabolic functions of OXT are related to its direct effect on adipose to decrease body weight gain and increase adipose tissue lipolysis and fatty acid beta-oxidation as well as to reduce glucose intolerance and insulin resistance [178]. Consistently, subchronic treatment of rats with OXT results in improved adipocyte differentiation and increased gene expression of factors involved in adipogenesis. This effect is related to increases in fatty acid binding protein, peroxisome proliferator-activated receptor gamma, insulin-sensitive glucose transporter 4, leptin, and CD31 mRNA levels [179]. 

Metabolic effects of OXT are closely related with several peripheral hormones. For instance, OXT can strengthen the satiety effect of CCK and bombesin-related peptides [88]. Adiponectin, hormone derived from adipocytes, hyperpolarizes OXT neurons in the PVN to modulate energy homeostasis and autonomic function. Thus, adiponectin plays specific roles in controlling the excitability of OXT neurons in regulating metabolic activity [180]. As OXT can dramatically change the energy balance, it is predictable that OXT could effectively modulate the pathogenesis of diabetes mellitus, atherosclerosis, and other metabolic diseases.

## 11. Concluding Remarks

Reviewing the functions of OXT and interactions between OXT and its targets, the most important actions of oxytocin are not at its effects on individual physiological processes but making whole organism in a fitting condition. This fitting function allows the body to maximize its potential to meet the demand of physiological processes and dismiss the adverse insults. We have no doubt in that properly using OXT can treat and even cure many central and peripheral diseases, while inappropriate OXT secretion can also be corrected through regulating the activity of its peripheral modulators, such as the olfactory bulb. Nevertheless, mechanisms underlying these functions remain to be further explored based on what we have known from genomic processes [181], neurochemical regulation of oxytocin neuronal activity [105, 182, 183], and OXTR signaling [7, 184], to behavioral establishment [185, 186]. We are full of confidence that, with further study of OXT functions and its regulation, OXT therapy will be in the spotlight again in our exploration of the approaches to enhance the quality of human life [187].
